# E-cigarettes and equity: a systematic review of differences in awareness and use between sociodemographic groups

**DOI:** 10.1136/tobaccocontrol-2016-053222

**Published:** 2016-12-21

**Authors:** Greg Hartwell, Sian Thomas, Matt Egan, Anna Gilmore, Mark Petticrew

**Affiliations:** 1 Department of Health Services Research and Policy, London School of Hygiene & Tropical Medicine, NIHR School for Public Health Research, London, UK; 2 Department for Health, University of Bath, UK Centre for Tobacco and Alcohol Studies (UKCTAS), Bath, UK

**Keywords:** Disparities, Electronic nicotine delivery devices, Socioeconomic status, Priority/special populations

## Abstract

**Objective:**

To assess whether electronic cigarette (e-cigarette) awareness, ‘ever use’ and current use vary significantly between different sociodemographic groups.

**Design:**

Systematic review.

**Data sources:**

Published and unpublished reports identified by searching seven electronic databases (PubMed, MEDLINE, Web of Science, EMBASE, Global Health, PsycINFO, CINAHL Plus) and grey literature sources.

**Study selection:**

Systematic search for and appraisal of cross-sectional or longitudinal studies that assessed e-cigarette awareness, ‘ever use’ or current use, and included subgroup analysis of 1 or more PROGRESS Plus sociodemographic groups. No geographical or time restrictions imposed. Assessment by multiple reviewers, with 17% of full articles screened meeting the selection criteria.

**Data extraction:**

Data extracted and checked by multiple reviewers, with quality assessed using an adapted tool developed by the Joanna Briggs Institute.

**Data synthesis:**

Results of narrative synthesis suggest broadly that awareness, ‘ever use’ and current use of e-cigarettes may be particularly prevalent among older adolescents and younger adults, males, people of white ethnicity and—particularly in the case of awareness and ‘ever use’—those of intermediate or high levels of education. In some cases, results also varied within and between countries.

**Conclusions:**

E-cigarette awareness, ‘ever use’ and current use appear to be patterned by a number of sociodemographic factors which vary between different countries and subnational localities. Care will therefore be required to ensure neither the potential benefits nor the potential risks of e-cigarettes exacerbate existing health inequalities.

## Introduction

Electronic cigarettes (e-cigarettes) are battery-powered devices which heat a liquid solution, usually containing nicotine, into an aerosol or ‘vapour’. Such products have proven attractive to many smokers given that they mimic the behavioural aspects of smoking and can deliver nicotine while avoiding the vast majority of toxins produced by the combustion of tobacco (the predominant risk factor for smoking-related disease).^1^

E-cigarette use has increased rapidly over recent years. In Great Britain, for instance, there are an estimated 2.8 million adults currently using them (6% of the adult population).^2^ However, despite such rapid uptake and their corresponding public profile, major research questions remain in relation to their true effectiveness as aids for quitting smoking and to possible health outcomes arising from sustained ‘vaping’.^3^ For instance, the authors of a recent Cochrane review found only two trials that followed participants for at least 6 months, rating their confidence in the evidence as low by GRADE standards.^4^ Very little is also known about variations in e-cigarette awareness and use between different sociodemographic groups; in other words, how these outcomes are patterned across society. The need for such equity-focused analyses is particularly pressing in light of evidence that smoking is significantly more common in the lowest income groups and is the leading cause of health inequalities.^5^ Although it has been argued that e-cigarettes could reduce inequalities,^6^ most tobacco control interventions exacerbate them (only tobacco tax has been shown to reduce inequalities),^7^ ^8^ and evidence on diffusion of innovations suggests that early adopters tend to be more affluent than other groups.^9^ This raises the possibility that if e-cigarettes prove effective at enabling quitting, they may in fact further widen—rather than reduce—inequalities in smoking.

Reviews in this area have largely considered overall population levels of awareness and use, without drilling down to analyse subgroups,^10^ or have been limited to compositional chemical safety issues.^11^

Our review therefore aimed to provide the first comprehensive assessment of whether e-cigarette awareness, ‘ever use’ and current use varied significantly across different sociodemographic groups.

## Methods

A full protocol for this systematic review was developed a priori and is registered with the PROSPERO international prospective register of systematic reviews (ID: CRD42015024163) at http://www.crd.york.ac.uk/PROSPERO.

### Search strategy

We searched seven databases (PubMed, MEDLINE, Web of Science, EMBASE, Global Health, PsycINFO, CINAHL Plus) for cross-sectional or longitudinal studies reporting on e-cigarette awareness, ‘ever use’ or current use. No search limits were set on study design (other than excluding intervention studies; see below), characteristics of participants or language of publication, but only studies published from 2006 onwards were retrieved, reflecting the nascence of viable e-cigarette markets around the world. Given research into these relatively novel devices is currently still limited, we were able simply to restrict our search syntax to synonyms for e-cigarettes, without requiring further search filters, thus reducing the risk of missing relevant studies. We also undertook a search of 12 grey literature databases and key websites. Further details of the search strategy are available in the online supplementary material file.

10.1136/tobaccocontrol-2016-053222.supp1 supplementary data

### Study selection and inclusion criteria

We included cross-sectional or longitudinal quantitative studies that reported at least one of the following outcomes: e-cigarette awareness, ‘ever use’ and current use. Studies predominantly defined awareness as having heard of e-cigarettes, ‘ever use’ as having tried an e-cigarette at least once in a respondent's lifetime and current use as having used e-cigarettes within the past 30 days. We included studies that used any form of summary measure for the included outcomes. Other than the aforementioned 2006 cut-off, there were no temporal or geographical restrictions: studies with international, national or subnational populations were included. Included studies had to sample both e-cigarette users and non-users, and needed to include subgroup analysis by one or more PROGRESS Plus sociodemographic group (PROGRESS Plus is an established taxonomy for classifying sociodemographic differences, with ‘PROGRESS’ standing for place of residence, race, occupation, gender, religion, education level, socioeconomic status and social capital, while ‘Plus’ represents additional categories such as age, disability and sexual orientation).^12^ We excluded intervention studies (due to our focus on real-world behaviour) and studies whose samples were restricted to e-cigarette users (due to a lack of information in such studies about the wider population these users were drawn from) or to patient populations (due to these samples not being directly comparable to other general population studies in our review).

After references that were obviously irrelevant had been removed, abstracts and titles of potentially relevant studies were independently screened against the eligibility criteria by one of two reviewers, who also both screened a 10% sample of each other's exclusion decisions. The full texts of all remaining studies were then obtained and assessed independently by two reviewers. Any discrepancies at each of these stages were resolved through discussion between the two reviewers, and with a third reviewer as required.

### Data extraction and risk of bias assessment

Following piloting of a data extraction form, one of two reviewers extracted data and assessed the risk of bias for each included study. Each reviewer then conducted their own assessment of risk of bias for all of the other reviewer's studies, and repeated the data extraction for a 25% sample of these. Discrepancies were resolved through discussion with a third reviewer. Data on the following factors were extracted: country, setting, population, study design, sampling methods, sample size, response rate, outcome measures reported and demographic subgroup analyses undertaken.

Risk of bias was assessed by two reviewers, adapting a tool developed by the Joanna Briggs Institute (JBI) specifically for studies of prevalence.^13^ We summarised risk of bias using the resulting 12 criteria and rated each study as high-quality, medium-quality or low-quality evidence depending on how many criteria were met.^i^

We extracted the available outcome measures on e-cigarette awareness, ‘ever use’ and current use, including the results of statistical tests (95% CIs or p values) for sociodemographic subgroup differences, where authors reported them. Studies which did not report any such statistical tests—and therefore provided only very weak evidence—were still included, for several reasons: in the case of one PROGRESS Plus group (occupation), the only evidence of any kind available came from such a study; in some circumstances, such studies were the only ones from a particular setting or country; and a sensitivity analysis showed that removing these types of studies did not materially affect the overall conclusions of the review.

In the narrative synthesis we undertook, we presented results in terms of relative differences in our outcomes between sociodemographic groups and summarised findings in an adapted effect direction plot.^14^ Meta-analyses were not possible given the heterogeneity of study designs (longitudinal, cross-sectional and repeat cross-sectional), settings (35 different countries), populations (studies often focused, for instance, on specific age groups), outcome measures (particularly for current use) and delineations of PROGRESS Plus subgroups (for instance, the different spatial categories for ‘place of residence’), as well as the lack of reported CIs within some studies. Providing point estimates for worldwide differences in awareness and use would also have been meaningless and potentially misleading for anyone seeking to use the results of the review to inform local or national action. Textual summaries therefore sought to elucidate the complexity and breadth of the data. This narrative synthesis of the results used the labels ‘high-quality', ‘medium-quality’ and ‘low-quality’ evidence, based on the aforementioned risk of bias assessment. The studies providing better quality evidence were emphasised by giving them prominence in our results summaries; low-quality evidence was reported, particularly where it was the only evidence available, but it was treated with caution. Summary findings reported in the Discussion section were based on any clear patterns emerging from the high-quality and medium-quality evidence. Study quality was also tabulated in the effect direction plot (see online supplementary table).

## Results

**Figure 1:**
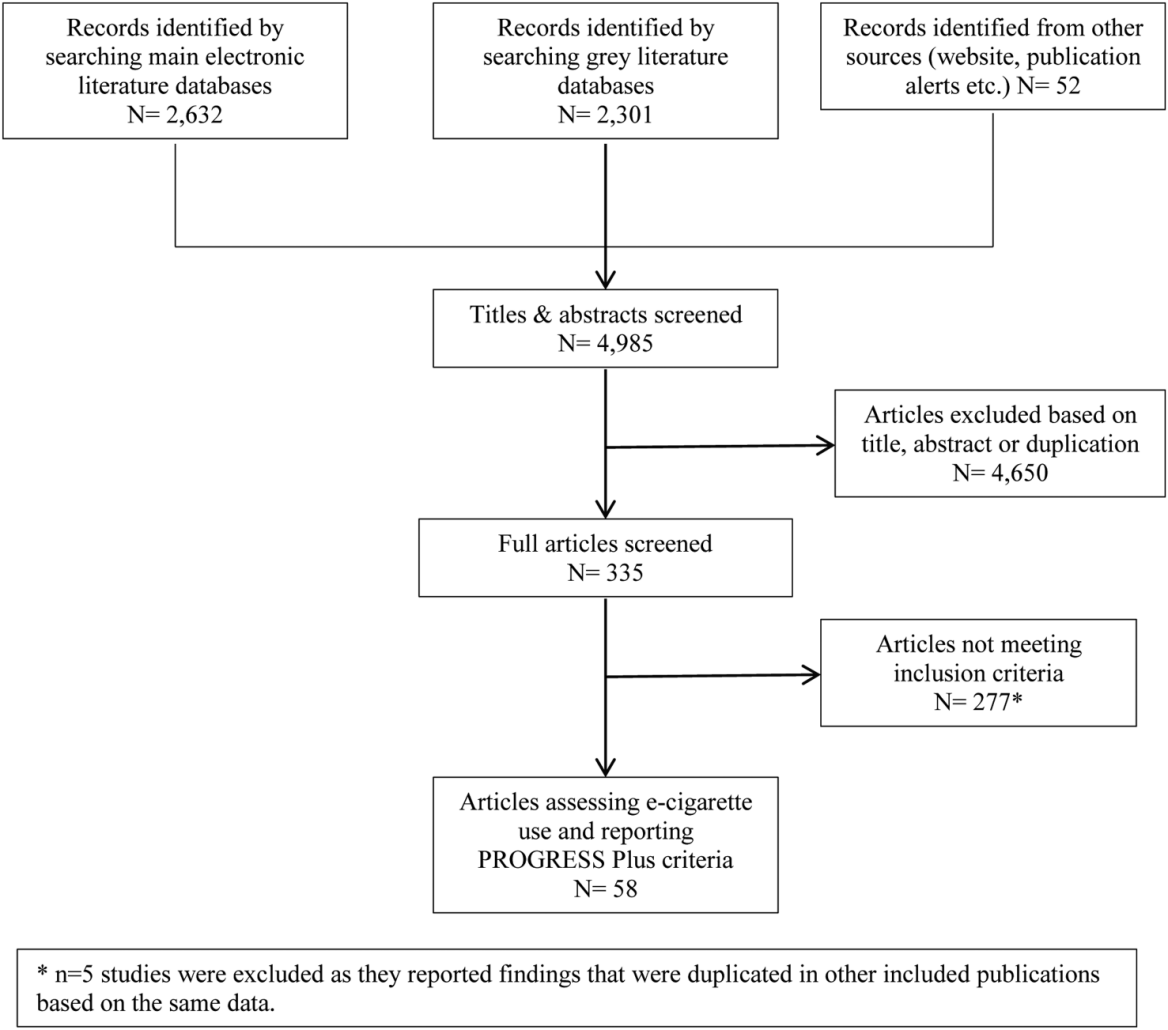
PRISMA flow chart.

We screened 4985 references and assessed the full text of 335 documents (figure 1). Fifty-eight studies from countries worldwide met our inclusion criteria: six longitudinal studies, 47 cross-sectional surveys and five repeat cross-sectional surveys. Twenty-one of these studies reported on awareness of e-cigarettes, 43 on ‘ever use’ and 32 on current use (see online supplementary table). Sample sizes reported ranged from 184 to 79 202 and were drawn from 35 nations around the world (all high-income countries). All studies used self-reported outcome measures that were of unknown validity or reliability due to the lack of research to date on such measures.

### Place of residence

Sixteen studies reported subgroup analysis by place of residence, with 8 studies reporting this for the outcome of e-cigarette awareness, 10 for ‘ever use’ and nine for current use. Only one study was rated as high-quality evidence, eight as medium quality and seven as low quality. Overall, while some differences were observed, no consistent themes emerged across the high-quality and medium-quality studies, perhaps because of the very varied countries and subregions sampled.

The highest quality study found no significant difference in e-cigarette awareness between urban and rural teenage boys in the USA,^15^ while two medium-quality European studies found higher ‘ever use’ in urban areas compared with rural ones.^16^ ^17^ One of these also reported on current use, finding this same urban > rural relationship existed for that outcome in Poland,^17^ as it did in a medium-quality 2014 study of South Korean high school students.^18^ The medium-quality 2013 International Tobacco Control (ITC) Four-Country Survey showed awareness and ‘ever use’ of e-cigarettes was higher in the countries where e-cigarettes were legal (USA and UK) than those where they were banned (Canada and Australia), though—interestingly—similar differences in current use were not observed between the countries.^19^

### Race/ethnicity

Twenty-eight studies reported subgroup analysis by race or ethnicity, with nine studies reporting this for the outcome of e-cigarette awareness, 19 for ‘ever use’ and 14 for current use. Three studies were rated as high-quality evidence, eight as medium and 17 as low. Overall, the most consistent findings from the better quality studies related to evidence of greater e-cigarette awareness and use among white populations compared with other ethnic groups. Almost all studies reporting this outcome came from the USA.

The 2013 ITC Four-Country Survey showed overall higher awareness among white/English-speaking adult smokers than non-white/non-English-speaking ones.^19^ This finding of higher awareness among respondents of white ethnicity was echoed across adult and teenage samples in all but one of the higher quality and medium-quality studies that examined the outcome in the USA.^15^ ^20–24^ For ‘ever use’, this same association with white ethnicity existed in four out of the five higher quality and medium-quality studies reporting significant differences,^20^ ^25–27^ while there was no such clear pattern of findings among the lower quality studies. Fewer studies reported on current use, but two of the three (medium and low quality) general population samples of adults in the USA also found higher current use in white respondents than those of various other ethnic groups.^23^ ^28^ A number of other studies of adults reported no significant differences between ethnicities for ‘ever use’,^23^ ^29–34^ or for current use.^27^ ^33^ ^35–38^

### Occupation

Only one study attempted subgroup analysis by occupation: a low-quality 2013 European Union (EU) survey that reported on awareness.^39^ Although statistical tests were not reported, the data suggested retired people might be less likely than other groups to be aware of e-cigarettes.

### Gender

Forty-six of the included studies reported subgroup data on gender, with 15 studies reporting this for the outcome of e-cigarette awareness, 34 for ‘ever use’ and 24 for current use. Four studies were rated as high-quality evidence, 12 as medium and 32 as low. Overall, all three outcomes were more prevalent among male respondents in many of the high-quality and medium-quality studies.

In all seven of the studies that reported statistically significant differences in awareness between males and females—which were mostly from the USA and four of which were high or medium quality—this outcome was higher in males.^19^ ^20^ ^22^ ^30^ ^33^ ^40^ ^41^ The two high-quality studies that reported on ‘ever use’ found no significant gender differences between children in Wales or adult smokers in the USA.^31^ ^42^ However, of the medium-quality studies that reported significant differences, four out of five samples (from the USA and Poland) found ‘ever use’ to be greater among males,^17^ ^23–26^ and three out of four (from the USA, Poland and South Korea) found the same to be the case for current use.^17^ ^18^ ^23^ ^24^ Several other studies found no significant differences for gender.

### Education level

Twenty-six studies in the review reported subgroup data on education level, with nine studies reporting this for the outcome of e-cigarette awareness, 18 for ‘ever use’ and 13 for current use. Three studies were rated as high-quality evidence, seven as medium and 16 as low. There was a broad pattern among the higher quality research of awareness and use (particularly ‘ever use’) being positively associated with higher levels of educational attainment.

Of the nine studies reporting on awareness (mainly from the USA), seven found statistically significant differences and, in each case, awareness was higher in subgroups with a greater level of educational attainment.^15^ ^19^ ^21–23^ ^30^ ^33^ ^40^ ^41^ For ‘ever use’, the two high-quality studies (both involving samples of adult smokers in the USA) also found the least ‘ever use’ in the groups with the lowest educational attainment,^20^ ^31^ while findings were more mixed in the medium-quality and low-quality studies, where around half of the studies reported no statistically significant differences between subgroups.^19^ ^23^ ^25^ ^27^ ^29^ ^30^ ^43–45^ For current use, the ITC Four-Country Survey found that, overall, participants with higher levels of educational attainment were more likely to report current use,^19^ while another medium-quality study (from the USA) found the inverse.^23^ Low-quality studies tended to report higher current use in the least educated groups,^23^ ^28^ ^33^ ^36^ or find no significant results.^27^ ^40^ ^45–47^

### Socioeconomic status

Twenty-three studies reported subgroup analysis by socioeconomic status (SES) of respondents, with five reporting this for the outcome of e-cigarette awareness, 18 for ‘ever use’ and seven for current use. Two studies were rated as high-quality evidence, seven as medium and 14 as low. Overall, no clear patterns emerged in studies reporting SES data.

The medium-quality 2013 ITC Four-Country Survey found that higher income participants were more likely to report awareness and ‘ever use’ in the USA, UK, Australia and Canada.^19^ However, none of the other high-quality and medium-quality studies found any statistically significant differences between different SES groups for any of the three outcomes,^16^ ^21^ ^22^ ^25^ ^26^ ^31^ ^42^ ^48^ with the exception of a 2014 South Korean study indicating higher current use among more affluent high school students.^18^ Lower quality studies tended to find mixed or non-significant results.^16^ ^25^ ^26^ ^28^ ^29^ ^31^ ^42^ ^43^ ^48–53^

### Disability or health status

Only four studies reported data on disability or health status related to our outcomes, with one study rated as high-quality evidence, two as medium and one as low. The one study to report on awareness—a medium-quality 2014 national survey of adults from the USA—found no significant differences by health status.^21^ For ‘ever use’, a high-quality 2012 national US study of adult smokers found that better self-reported health status was associated with this outcome,^20^ while a medium-quality 2014 study of adult current and former smokers from eight US ‘market areas’ found no significant differences.^25^ The one (low-quality 2014 US) study to report on current use found this to be associated with medical illnesses, greater depressed mood and greater alcohol use.^37^

### Sexual orientation

Only two studies reported data on sexual orientation related to our outcomes, with one of these rated as medium-quality evidence and the other rated as low. The medium-quality study—a 2014 online survey of adults from the USA—found that awareness was not associated with sexual orientation.^22^ The low-quality 2014 survey from the USA found higher rates of current use in lesbian, gay and bisexual respondents compared with respondents who were heterosexual or did not specify a sexual orientation.^28^

### Age

Forty-eight studies reported subgroup analysis by age of respondents, with 18 reporting this for the outcome of e-cigarette awareness, 38 for ‘ever use’ and 22 for current use. Three studies were rated as high-quality evidence, 11 as medium and 34 as low. The overall direction of evidence pointed to older adolescents and young adults driving levels of awareness and use of e-cigarettes: findings from all the higher quality studies and many of the other studies fitted this pattern.

High-quality and medium-quality studies with samples from the USA, UK, Canada, Australia and Italy showed greater awareness in older adolescents compared with younger children, and in younger adults compared with older ones.^15^ ^19–21^ ^24^ ^40^ Throughout the high-quality and medium-quality studies which identified statistically significant differences for ‘ever use’ (10 studies from the aforementioned five countries, plus Poland and the EU more widely),^16^ ^17^ ^19^ ^20^ ^23–25^ ^27^ ^31^ ^40^ and for current use (4 studies from Italy, USA, South Korea and Poland),^17^ ^18^ ^24^ ^40^ these outcome measures were again greatest in older children and younger adults. Lower quality studies found fewer significant differences, often lacking sufficient statistical analysis, but those that did were virtually unanimous in observing the same patterns of higher use in older adolescents and younger adults.^28–30^ ^36^ ^43–45^ ^51^ ^53–55^

## Discussion

### Principal findings

We systematically reviewed both published and grey literature for studies reporting sociodemographic differences in e-cigarette awareness, ‘ever use’ and current use. We found variability in social patterning across all outcomes, but have drawn attention to findings that tend to recur in the high-quality and medium-quality studies. Across all the outcomes, we found that e-cigarettes appear to have achieved greater reach among older adolescents and younger adults, males and people of white ethnicity. For awareness and ‘ever use’, this was also the case for subpopulations with relatively higher educational attainment. Studies varied in how they defined these characteristics. For the other PROGRESS Plus characteristics we examined, findings were too inconsistent to enable us to identify a pattern supported by higher quality evidence, and in the case of sexual orientation, disability/health status and occupation the evidence base is still very small. The only previous review to investigate a related research question included 23 studies and did not incorporate any quality assessment.^56^ Hence, studies with conflicting findings were synthesised without reference to the direction of effects suggested by the best available evidence. That review did not identify studies that found distinct patterns of use across racial/ethnic groups, which the authors suggested could have been due to included studies being underpowered to test this association. It reported conflicting evidence relating to e-cigarette use when comparing subpopulations with different educational levels. In common with our review, it highlighted greater use among young adults.

### Strengths and limitations

We have followed Cochrane guidance and PRISMA reporting standards for systematic reviews. An extensive search was performed of published and grey literature from the first seven years that e-cigarette markets have been expanding throughout the developed world (2006 to October 2014, when our searches were run). There are, however, limitations to our study. Our review did not explore e-cigarette use in specific clinical populations (we focused on general population samples to ensure we were comparing like-for-like as far as possible). However, the best way of demonstrating links between, for instance, mental illness and e-cigarettes would arguably be through any general population samples that performed subgroup analysis by mental health status. Our inclusion criteria would have included such studies (under the health status/disability PROGRESS Plus subgroup), but none showed up in our various database searches. While our quality assessment was based on an established tool for prevalence studies,^13^ the tool has been tailored to our requirements for this review and these adaptations are not validated. In addition, we appraised studies with reference to our specific review question; a study could in theory use robust methods for addressing its own research question but less robust methods for addressing the reviews. Like all reviews, we were limited by the evidence available and its reporting. For instance, most studies reporting current use defined this as any use of e-cigarettes within the past 30 days, which might have included some people who had simply tried e-cigarettes recently rather than become regular users. Unfortunately, there were insufficient studies using a tighter definition to enable us to assess the sociodemographic determinants of strictly regular e-cigarette use. There is of course also a risk that publication bias may exist, in which studies with non-significant findings in relation to awareness and use may be less likely to be published. However, the large proportion of studies in the review reporting non-significant findings—and the fact that these were often smaller studies and often fell into the lower quality of evidence category—may indicate that this bias is unlikely to be exerting a major influence on our review. Similarly, despite our wide-ranging searches, no eligible low-income or middle-income country studies came up in our trawls. There is no clear way of assessing the degree to which this reflects a bias in the body of research that has been conducted versus any bias in the databases we searched. Our databases certainly will have had an English language and high-income country bias, but it is also probable that there is not yet any significant volume of research on e-cigarettes from low-income and middle-income countries—like all new technologies, e-cigarettes will have spread much more quickly among high-income country markets initially. Finally, we took the decision not to include smoking of conventional cigarettes as a variable for analysis. Doing so would have pulled in an extremely large amount of data not directly relevant to our research question and introduced further heterogeneity (given differences in how smoking status was recorded across studies). While it might have allowed us to analyse issues such as the ‘gateway’ hypothesis that young non-smokers may be moving from e-cigarettes on to tobacco, such questions are already being addressed effectively through other research more directly focused on this area.^1^ ^6^

### Implications for research, policy and practice

While e-cigarettes are widely assumed to be safer than combustible tobacco, the long-term health impacts of vaping are as yet unknown. It is therefore important to understand how far e-cigarette familiarity and adoption vary between different social groups, since this can inform monitoring work to ensure any risks from e-cigarettes do not widen existing health inequalities. Conversely, studies such as these can also help ensure any opportunities offered by e-cigarettes as aids for quitting smoking are distributed fairly across society. The fact that younger and more educated groups may have been particularly likely to trial e-cigarettes is of course not a cause for concern in itself, since this is a common pattern among early adopters of technologies generally.^9^ However, greater future research focusing specifically on e-cigarette users who have successfully quit smoking would be valuable in helping to monitor any inequality implications. It would be useful, for instance, to understand whether these e-cigarette users, like smokers generally, are more likely to be from lower socioeconomic groups or not. Similarly, future studies should stratify their findings by relevant sociodemographic groups, to ensure that further subgroup analyses are possible, and should consider more precise measures of current use to capture this concept more accurately. For instance, the US Centre for Disease Control (CDC) defines ‘frequent smoking’ as smoking cigarettes on 20 or more days out of the past 30.^57^ Further studies could adopt this as an easily understandable metric for e-cigarette use, which would additionally allow for useful comparisons with US studies. More precision around the specific types of e-cigarettes being used—and particularly whether they contain nicotine or not—would also be valuable (few studies in our review asked respondents about this), as would a review specifically focusing on studies involving samples of particular patient groups, since these were excluded from our study. Finally, future research should be sensitive to the fact that increasing studies will be emerging from middle-income and low-income countries as e-cigarettes gain further traction in those markets.

We have not reviewed findings on quitting/uptake of smoking, dual usage of e-cigarettes and traditional combustible cigarettes, or health outcomes. Some of these research questions will, we assume, be addressed over time as e-cigarette research begins to consider medium-term and long-term outcomes. Indeed, this review should help lay the foundations for effective public health action in this area. While we must wait for evidence of longer term impacts of e-cigarettes to accumulate, this study provides a baseline early indication of the reach these products have established among different population subgroups. It thus provides an essential first step towards monitoring the population and health inequality impacts of e-cigarettes with more clarity and granularity as these technologies diffuse through societies.

## Conclusions

E-cigarette awareness, ‘ever use’ and current use appear to be patterned by a number of sociodemographic factors. While the evidence is frequently inconsistent, our review has allowed us to identify older children, younger adults, males and people of white ethnicity as the groups more likely to be aware of, to have ever used and to currently use these products. Awareness and ever use also appear to be greater in people with relatively higher educational levels. This study thereby highlights the importance, in research and practice, of carefully recording sociodemographic determinants of e-cigarette use and potential outcomes of such use (quitting or uptake of smoking, as well as health outcomes) to ensure that e-cigarettes do not widen existing health inequalities.

**What this paper adds**

- Electronic cigarette (e-cigarette) awareness, ‘ever use’ and current use are patterned by a number of sociodemographic factors.
- These outcomes appear to be most prevalent in older children, younger adults, males, people of white ethnicity and—particularly for awareness and ‘ever use’—those with relatively higher educational levels.
- Further attention may be required to ensure neither the potential benefits nor the potential risks of e-cigarettes are allowed to increase existing health inequalities.

## Footnotes

i
supplementary appendices with full details on the definitions used for the high, medium and low categories are available from the authors.
