# Supplementary material for: E-cigarettes and equity: a systematic review of differences in awareness and use between sociodemographic groups
Source: Tob Control. 2016 Dec 21;26(e2):e85–91. doi: 10.1136/tobaccocontrol-2016-053222 (PMC5739861; doi:10.1136/tobaccocontrol-2016-053222)
Supplement: supplementary data [file tobaccocontrol-2016-053222supp001.pdf]

<u>Supplementary Table: Effect Direction Plot</u>

Ordered by PROGRESS Plus sub-group criteria, and then grouped by evidence quality

Key: ● = outcome reported, statistically significant difference found; ○ = outcome reported, no statistically significant difference found;

◌ = outcome reported, no indication of any test for statistical significance; [blank box] = outcome not studied

Lead Author	Country	lityEvidence	Sample	Aware	Ever use	Current Use	Key findings
**PLACE OF RESIDENCE**							
Pepper 2013 (1)	USA	H	421 male adolescents	○			
Adkison 2013 (2)	UK, USA, Canada, Australia	M	5,939 adult smokers	●	●	○	USA & UK > Australia & Canada (awareness); USA & UK > Canada (ever use); USA > Australia (ever use)
Gallus 2014 (3)	Italy	M	3,000 adults	●	○	●	Northern Italy & Central Italy > Southern Italy (awareness); Southern Italy > Central Italy (current use)
Giovenco 2014 (4)	USA	M	2,136 current/recent smokers		○	○	
Goniewicz 2012 (5)	Poland	M	20,240 high school/uni students		●	●	Urban > rural
King 2014 (6)	USA	M	14,758 adults	○	○	○	
Lee 2014 (7)	South Korea	M	75,643 high school students			●	City & metropolitan city > province
Tan 2014 (8)	USA	M	9,080 adults	○			
Vardavas 2014 (9)	EU member states	M	26,566 adults	●	●		Individual countries: various international differences (awareness & ever use); Large towns > rural (ever use); North & East Europe > West Europe (ever use)
Agaku 2014 (10)	EU member states	L	Adults in 25 countries			●	Individual countries: various international differences [a]
Agaku 2014 (11)	USA	L	60,192 adults			○	
Douptcheva 2013 (12)	Switzerland	L	5,081 men		●		German region > French region [b]
McMillen 2012 (13)	USA	L	3,240 adults		○		
Pepper 2014 (14)	USA	L	17,522 adults	○			
Popova 2013 (15)	USA	L	7,776 adult current/recent smokers		○		
Regan 2013 (16)	USA	L	41,240 adults	○	○	○	
**RACE / ETHNICITY**							
Kasza 2013 (17)	USA	H	6,110 adult smokers		○		
Pearson 2012 (18)	USA	H	19,026 adult smokers	●	●		White > African American
Pepper 2013 (1)	USA	H	421 male adolescents	●			Other races > Hispanic
Adkison 2013 (2)	UK, USA, Canada, Australia	M	5,939 adult smokers	●	○	○	White/English > Non-white/Non-English [c]

Lead Author	Country	alityEvidence	Sample	Aware	Ever use	Current Use	Key findings
Amrock 2014 (19)	USA	M	24,658 high school students	●	●	●	White, Mixed/Multiple race > Black (awareness); Hispanic > Asian (ever & current use) [d]
Emery 2014 (20)	USA	M	17,522 adults	●			White > Hispanic, Other
Giovenco 2014 (4)	USA	M	2,136 adult current/recent smokers		●	○	White > Other races (ever use) [e]
King 2014 (6)	USA	M	14,758 adults	●	○	●	White > Black (awareness); White > Hispanic (current use) [f]
Lippert 2014 (21)	USA	M	15,264 adolescents		●		Other races > Black, Mexican-American
Richardson 2014 (22)	USA	M	1,487 adult current/former smokers		●		White > Hispanic
Tan 2014 (8)	USA	M	9,080 adults	●			White > Hispanic
Agaku 2014 (11)	USA	L	60,192 adults			●	'Other, Non-Hispanic', White > Other races [d,g]
Camenga 2014 (23)	Connecticut & NY, USA	L	3,102 high school students			◌	
Camenga 2014 (24)	Connecticut & NY, USA	L	4,766 high school students			●	Mixed results
Choi 2013 (25)	Minnesota, USA	L	2,624 adults	○	○		
Czoli 2014 (26)	Canada	L	1,188 young adults		○	○	
Grana 2014 (27)	USA	L	1,549 adult smokers			○	
Hayes 2014 (28)	USA	L	2,376 adult smokers			○	
Hyland 2013 (29)	New York, USA	L	2 schools		○	○	
Li 2013 (30) [h]	New Zealand	L	840 adult current/recent smokers		○		
McMillen 2012 (13)	USA	L	3,240 adults		○		
Pokhrel 2014 (31)	Hawaii, USA	L	307 undergrad students		●	●	Filipino > White
Pokhrel 2013 (32)	Hawaii, USA	L	1,685 adult smokers		●		White > Native Hawaiian
Popova 2013 (15)	USA	L	7,776 adult current/recent smokers		●		Asian > White
Regan 2013 (16)	USA	L	41,240 adults	●	○	○	White > African, Hispanic
Sherratt 2014 (33)	Liverpool, England	L	256 adult smokers			○	
Sutfin 2013 (34)	North Carolina, USA	L	4,857 undergrad students		●		Hispanic, Other > White
Vickerman 2013 (35)	USA	L	7,966 adult smokers		●		White > Other races
**OCCUPATION**							
EU 2012 (36)	27 EU member states	L	26,751 adults	◌			
**GENDER**							
CHETS 2014 (37)	Wales	H	1,601 children		○		
Kasza 2013 (17)	USA	H	6,110 adult smokers		○		
Pearson 2012 (18)	USA	H	19,026 adult smokers	●			Men > Women
Pepper 2013 (1)	USA	H	421 male adolescents	○			
Adkison 2013 (2)	UK, USA, Canada, Australia	M	5,939 adult smokers	●	○	○	Men > Women [c]
Amrock 2014 (19)	USA	M	24,658 high school students	○	●	●	Men > Women [d]
Emery 2014 (20)	USA	M	17,522 adults	●			Men > Women
Gallus 2014 (3)	Italy	M	3,000 adults	●	○	○	Men > Women
Giovenco 2014 (4)	USA	M	2,136 adult current/recent smokers		○	○	

Lead Author	Country	lityEvidence	Sample	Aware	Ever use	Current Use	Key findings
Goniewicz 2012 (5)	Poland	M	20,240 high school/uni students		●	●	Males > Females
King 2014 (6)	USA	M	14,758 adults	○	●	●	Women > Men [f]
Lee 2014 (7)	South Korea	M	79,202 high school students			●	Males > Females
Lippert 2014 (21)	USA	M	15,264 adolescents		●		Men > Women
Richardson 2014 (22)	USA	M	1,487 adult current/former smokers		●		Men > Women
Tan 2014 (8)	USA	M	9,080 adults	○			
Vardavas 2014 (9)	EU member states	M	26,566 adults		○		
Agaku 2014 (11)	USA	L	60,192 adults			●	Men > Women [d,g]
Agaku 2014 (10)	Multiple countries	L	Adults in 25 countries			●	Mixed results in different countries [a]
ASH Wales 2014 (38)	Wales	L	740 adolescents	○	○	○	
Biener 2013 (39)	USA	L	5,150 adults		○	○	
Brown 2014 (40)	UK	L	4,117 adult smokers		○		
Camenga 2014 (23)	USA	L	3,102 high school students			◌	
Cho 2011 (41)	Seoul, South Korea	L	4,341 middle/high school students		●		Males > Females
Choi 2013 (25)	Minnesota, USA	L	2,624 adults	●	●		Men > Women
Czoli 2014 (26)	Canada	L	1,188 young adults		○	○	
Dautzenberg 2013 (42)	Paris, France	L	3,409 high school students		○		
Dockrell 2013 (43)	UK	L	12,432 adults		○	○	
EU 2012 (36)	27 EU member states	L	26,751 adults	◌			
Grana 2014 (27)	USA	L	1,549 current/recent smokers			●	Women > Men [i]
Hyland 2013 (29)	New York, USA	L	2 schools		○	○	
Kinnunen 2013 (44)	Finland	L	3,535 adolescents	◌	◌		
Kralikova 2013 (45)	Czech Republic	L	1,738 smokers		●	●	Men > Women
Krishnan-Sarin 2014 (46)	Connecticut, USA	L	4,780 middle/high school students		●		Males > Females
Kvaavik 2014 (47)	Norway	L	629 adult current/former smokers			●	Men > Women [j]
Li 2013 (30) [h]	New Zealand	L	840 adult current/recent smokers		○		
Martinez-Sanchez 2014 (48)	Barcelona, Spain	L	1,291 adults		○	○	
McMillen 2012 (13)	USA	L	3,240 adults		○		
Pearson 2011 (49)	USA	L	3,638 adults	●	●		Men > Women
Pokhrel 2014 (31)	Hawaii, USA	L	307 undergrad students		○		
Pokhrel 2013 (32)	Hawaii, USA	L	1,685 adult smokers		○		
Popova 2014 (15)	USA	L	7,776 adult current/recent smokers		●		Women > Men
Regan 2013 (16)	USA	L	41,240 adults	●	●	○	Men > Women (aware); Women > Men (ever use)
Sherratt 2014 (33)	Liverpool, England	L	256 adult smokers			●	Females > Men
Sochor 2014 (50)	Brno city, Czech Rep	L	966 adults			●	Men > Women
Stillman 2014 (51)	USA	L	723 flight attendants			●	Men > Women [k]

Lead Author	Country	lityEvidence	Sample	Aware	Ever use	Current Use	Key findings
Sutfin 2013 (34)	North Carolina, USA	L	4,857 undergrad students		●		Men > Women
Vickerman 2013 (35)	6 states, USA	L	7,966 adult smokers		○		
WHO 2013 (52)	Qatar	L	8,398 adults	○	○	○	
**EDUCATION LEVEL**							
Kasza 2013 (17)	USA	H	6,110 adult smokers		●		Intermediate > lower education
Pearson 2012 (18)	USA	H	19,026 adult smokers		●		Higher > lower education
Pepper 2013 (1)	USA	H	421 male adolescents	○			
Gallus 2014 (3)	Italy	M	3,000 adults	●	●	○	Higher > lower education (aware); Intermediate > lower education (ever use)
Adkison 2013 (2)	UK, USA, Canada, Australia	M	5,939 adult smokers	●	○	●	Higher > lower education [c]
Emery 2014 (20)	USA	M	17,522 adults	●			Higher > lower education
Giovenco 2014 (4)	USA	M	2,136 adult current/recent smokers		○	○	
King 2014 (6)	USA	M	14,758 adults	○	○	●	Less than high school > High school [f]
Richardson 2014 (22)	USA	M	1487 adult current/former smokers		○		
Tan 2014 (8)	USA	M	9,080 adults	●			Higher > lower education
Agaku 2014 (11)	USA	L	60,192 adults			●	Lower > higher education [d,g]
Choi 2013 (25)	Minnesota, USA	L	2,624 adults	●	○		Higher > lower education
Czoli 2014 (26)	Canada	L	1,188 young adults		●	●	Lower > higher education (ever use); Higher > lower education (current use)
Douptcheva 2013 (12)	Switzerland	L	5,081 men		●		Secondary > tertiary or primary [b]
Grana 2014 (27)	USA	L	1,549 smokers			●	Lower > higher education
Li 2013 (30) [h]	New Zealand	L	840 adult current/recent smokers		○		
Martinez-Sanchez 2014 (48)	Barcelona, Spain	L	1,291 adults		○	○	
McMillen 2012 (13)	USA	L	3,240 adults		●		Intermediate > lower education
Pearson 2011 (49)	USA	L	3,638 adults	●	●		Higher > lower education
Pokhrel 2013 (32)	Hawaii, USA	L	1,685 adult smokers		○		
Popova 2013 (15)	USA	L	7,776 adult current/recent smokers		○	●	Intermediate > higher education
Regan 2013 (16)	USA	L	41,240 adults	●	●	●	Higher > lower education (aware); Lower > higher education (ever & current use)
Sherratt 2014 (33)	Liverpool, England	L	256 adult smokers			○	
Sochor 2014 (50)	Brno city, Czech Republic	L	966 adults			○	
Stillman 2014 (51)	USA	L	723 flight attendants			○	
Vickerman 2013 (35)	USA	L	7,966 adult smokers		●		Higher > lower education
**SOCIOECONOMIC STATUS**							
CHETS 2014 (37)	Wales	H	1,601 children		○		
Kasza 2013 (17)	USA	H	6,110 adult smokers		○		
Adkison 2013 (2)	UK, USA, Canada, Australia	M	5,939 adult smokers	●	●	○	Higher > lower income [c]
Emery 2014 (20)	USA	M	17,522 adults	○			
Lippert 2014 (21)	USA	M	15,264 adolescents		○		
Lee 2014 (7)	South Korea	M	79,202 high school students			●	Higher > lower weekly allowance

Lead Author	Country	alityEvidence	Sample	Aware	Ever use	Current Use	Key findings
Richardson 2014 (22)	USA	M	1,487 adult current/former smokers		○		
Tan 2014 (8)	USA	M	9,080 adults	○			
Vardavas 2014 (9)	EU member states	M	26,566 adults		○		
Agaku 2014 (11)	USA	L	60,192 adults			●	Lower > higher income [d,g]
Brown 2014 (40)	UK	L	4,117 adult smokers		●		Higher > lower SES [i]
Dockrell 2013 (43)	UK	L	12,432 adults		○	○	
Douptcheva 2013 (12) [b]	Switzerland	L	5,081 men		○		
King 2013 (53)	USA	L	10,739 adults	○	○		
Kinnunen 2013 (44)	Finland	L	3,535 adolescents		●		Mixed results
Li 2013 (30)	New Zealand	L	840 adult current/recent smokers		●		Higher > lower income [h]
Pokhrel 2014 (31)	Hawaii, USA	L	307 adult current/former/never smokers		○		
Pokhrel 2013 (32)	Hawaii, USA	L	1,685 adult smokers		○		
Popova 2013 (15)	USA	L	7,776 adult current/recent smokers		○	●	Lower > higher income
Regan 2013 (16)	USA	L	41,240 adults	○	●	○	Mixed results
Sochor 2014 (50)	Brno city, Czech Republic	L	966 adults			○	
Sutfin 2013 (34)	North Carolina, USA	L	4,857 undergrad students		○		
Vickerman 2013 (35)	USA	L	7,966 adult smokers		●		Mixed results
**DISABILITY OR HEALTH STATUS**							
Pearson 2012 (18)	USA	H	19,026 adult smokers		●		Better > worse self-reported health status
Richardson 2014 (22)	USA	M	1,487 adult current/former smokers		○		
Tan 2014 (8)	USA	M	9,080 adults	○			
Hayes 2014 (28)	USA	L	2,376 adult smokers			●	Medical illnesses, depressed mood, alcohol use > absence of these
**SEXUAL ORIENTATION**							
Emery 2014 (20)	USA	M	17,522 adults	○			
Agaku 2014 (11)	USA	L	60,192 adults			●	Lesbian, gay or bisexual > heterosexual & unspecified [d,g]
**AGE**							
Kasza 2013 (17)	USA	H	6,110 adult smokers		●		18-24yo > 40yo+
Pearson 2012 (18)	USA	H	19,026 adult smokers	●	●		Younger > Older adults
Pepper 2013 (1)	USA	H	421 male adolescents	●			Older > Younger children
Adkison 2013 (2)	UK, USA, Canada, Australia	M	5,939 adult smokers	●	●	○	Younger > Older adults [c]
Amrock 2014 (19)	USA	M	24,658 high school students	●	●	●	Older > Younger children [d]
King 2014 (6)	USA	M	14,758 adults	○	●	○	Younger > Older adults [f]
Gallus 2014 (3)	Italy	M	3,000 adults	●	●	●	Younger > Older adults
Giovenco 2014 (4)	USA	M	2,136 adult current/recent smokers		●	○	Younger > Older adults
Goniewicz 2012 (5)	Poland	M	20,240 high school/uni students		●	●	High school students > Uni students
Lee 2014 (7)	Korea	M	79,202 high school students		◌	●	Older > Younger children

Lead Author	Country	lityEvidence	Sample	Aware	Ever use	Current Use	Key findings
Lippert 2014 (21)	USA	M	15,264 adolescents		○		
Richardson 2014 (22)	USA	M	1,487 adult current/former smokers		●		Younger > Older adults
Tan 2014 (8)	USA	M	9,080 adults	●			Younger > Older adults
Vardavas 2014 (9)	EU member states	M	26,566 adults		●		Younger > Older adults
Agaku 2014 (11)	USA	L	60,192 adults			●	Younger > Older adults [d,g]
ASH 2014 (54)	Great Britain	L	53,719 adults & children	○	○	○	
ASH Wales 2014 (38)	Wales	L	740 adolescents	○	○	○	
Baeza-Loya 2014 (55)	USA	L	184 adults		○		
Biener 2014 (39)	USA	L	5,150 adults			○	
Brown 2014 (40)	UK	L	4,117 adult smokers		○		
Cho 2011 (41)	Seoul & province in Korea	L	4,341 middle/high school students		○		
Choi 2013 (25)	Minnesota, USA	L	2,624 adults	○	●		20-24year olds > 25-28year olds
Czoli 2014 (26)	Canada	L	1,188 young adults		○	○	
Dautzenberg 2013 (42)	Paris, France	L	3,409 high school students		○		
Dockrell 2013 (56)	UK	L	12,171 adolescents	○	○	○	
Dockrell 2013 (43)	UK	L	12,432 adults		●		Younger > Older adults
Douptcheva 2013 (12) [b]	Switzerland	L	5,081 men		○		
EU 2012 (36)	27 EU member states	L	26,751 adults	○			
Grana 2014 (27)	USA	L	1,549 adult smokers			●	Younger > Older adults
Johns 2014 (57)	USA	L	504 adolescents		●		Older > Younger children
King 2013 (53)	USA	L	10,739 adults	●	○		Younger > Older adults
Kinnunen 2013 (44)	Finland	L	3,535 adolescents	○	○		
Kralikova 2013 (45)	Czech Republic	L	1,738 smokers		●	●	Younger > Older adults (ever use); Older > Younger adults (current use)
Krishnan-Sarin 2014 (46)	USA	L	4,780 middle/high school students	○	○	○	
Li 2013 (30)	New Zealand	L	840 adult current/recent smokers		●		Younger > Older adults [h]
Martinez-Sanchez 2014 (48)	Barcelona, Spain	L	1,291 adults		●	○	Younger > Older adults
McMillen 2012 (13)	USA	L	3,240 adults		○		
Pepper 2014 (14)	USA	L	17,522 adults	●			Younger > Older adults
Pokhrel 2014 (31)	Hawaii, USA	L	307 undergrad students		●	●	Younger > Older adults
Pokhrel 2013 (32)	Hawaii, USA	L	1,685 adult smokers		●		Younger > Older adults
Popova 2013 (15)	USA	L	7,776 adult current/recent smokers		●		Younger > Older adults
Regan 2013 (16)	USA	L	41,240 adults	●	○	○	Younger > Older adults
Sherratt 2014 (33)	Liverpool, England	L	256 adult smokers			○	
Sochor 2014 (50)	Brno city, Czech Republic	L	966 adults			●	Older > Younger adults
Stillman 2014 (51)	USA	L	723 flight attendants			○	
Sutfin 2013 (34)	North Carolina, USA	L	4,857 undergrad students		○		

Lead Author	Country	lityEvidence	Sample	Aware	Ever use	Current Use	Key findings
Vickerman 2013 (35)	6 states, USA	L	7,966 adult smokers		○		
Yong 2014 (58)	Australia	L	~13,500 current/former smokers [l]	○	○		

[a] Current use defined as self-reported use ‘regularly’ or ‘occasionally’.
[b] Among smokers only. Ever use defined as any use in last 12 months.
[c] Plot shows results for all 4 countries combined.
[d] Statistical significance inferred by review authors from non-overlapping confidence intervals
[e] Rates also higher among whites for ‘established use’ (used an e-cigarette at least once in the past 30 days and more than 50 times in their lifetime).
[f] Study reports multiple years of data; for reporting purposes, plot reflects data from most recent year.
[g] Current use defined as reporting smoking electronic cigarettes at least once during their lifetime and now smoking them ‘every day’ or ‘some days’.
[h] Ever purchase of an e-cigarette taken as proxy for ever use.
[i] Among smokers only.
[j] Among both current and former smokers respectively.
[k] Current use defined as ‘do you use e-cigarettes?’ (i.e. no 30-day timeframe given).
[l] Estimated by review team due to sample size not being reported directly by authors.

1. Pepper JK, Reiter PL, McRee A-L, et al. Adolescent Males' Awareness of and Willingness to Try Electronic Cigarettes. *Journal of Adolescent Health.* 2013;52(2):144-50.
2. Adkison SE, O'Connor RJ, Bansal-Travers M, et al. Electronic nicotine delivery systems: international tobacco control four-country survey. *American journal of preventive medicine.* 2013;44(3):207-15.
3. Gallus S, Lugo A, Pacifici R, et al. E-Cigarette Awareness, Use, and Harm Perception in Italy: A National Representative Survey. *Nicotine Tob Res.* 2014;16(12):1541–1548.
4. Giovenco DP, Lewis MJ, Delnevo CD. Factors Associated with E-cigarette Use: A National Population Survey of Current and Former Smokers. *Am J Prev Med.* 2014;47(4):476-80.
5. Goniewicz ML, Zielinska-Danch W. Electronic Cigarette Use Among Teenagers and Young Adults in Poland. *Pediatrics*. 2012;130(4):E879-E85.
6. King BA, Patel R, Nguyen K, et al. Trends in Awareness and Use of Electronic Cigarettes among U.S. Adults, 2010-2013. *Nicotine Tob Res.* 2014;17,219-227.
7. Lee S, Grana RA, Glantz SA. Electronic cigarette use among Korean adolescents: a cross-sectional study of market penetration, dual use, and relationship to quit attempts and former smoking. *J Adolesc Health.* 2014;54(6):684-90.
8. Tan ASL, Bigman CA. E-Cigarette Awareness and Perceived Harmfulness Prevalence and Associations with Smoking-Cessation Outcomes. *American Journal of Preventive Medicine.* 2014;47(2):141-9.

9. Vardavas CI, Filippidis FT. Determinants and prevalence of e-cigarette use throughout the European Union: a secondary analysis of 26 566 youth and adults from 27 Countries. *Tob Control* 2015;24:442–8.

10. Agaku IT, Filippidis FT, Vardavas CI, et al. Poly-tobacco use among adults in 44 countries during 2008-2012: Evidence for an integrative and comprehensive approach in tobacco control. *Drug Alcohol Depend.* 2014 Jun 1;139:60-70.

11. Agaku IT, King BA, Husten CG, et al. Tobacco product use among adults--United States, 2012-2013. *MMWR Morb Mortal Wkly Rep.* 2014 Jun 27;63(25):542-7.

12. Douptcheva N, Gmel G, Studer J, et al. Use of electronic cigarettes among young Swiss men. *J Epidemiol Community Health.* 2013;67(12):1075-6.

13. McMillen R, Maduka J, Winickoff J. Use of emerging tobacco products in the United States. *Journal of environmental and public health.* 2012;2012:989474.

14. Pepper JK, Emery SL, Ribisl KM, et al. Hows U.S. adults find out about electronic cigarettes: implications for public health messages. *Nicotine Tob Res.* 2014;16(8):1140-4.

15. Popova L, Ling PM. Alternative tobacco product use and smoking cessation: a national study. *American journal of public health.* 2013;103(5):923-30.

16. Regan AK, Promoff G, Dube SR, et al. Electronic nicotine delivery systems: adult use and awareness of the 'e-cigarette' in the USA. *Tob Control.* 2013;22(1):19-23.

17. Kasza KA, Bansal-Travers M, O'Connor RJ, et al. Cigarette smokers' use of unconventional Tobacco products and associations with quitting activity: Findings from the ITC-4 U.S. cohort. *Nicotine Tob Res.* 2014;16(6):672–681.

18. Pearson JL, Richardson A, Niaura RS, et al. e-Cigarette awareness, use, and harm perceptions in US adults. *American journal of public health.* 2012;102(9):1758-66.

19. Amrock SM, Zakhar J, Zhou S, et al. Perception of E-cigarettes' Harm and Its Correlation With Use Among U.S. Adolescents. *Nicotine Tob Res.* 2015 Mar;17(3):330-6.

20. Emery SL, Vera L, Huang J, et al. Wanna know about vaping? Patterns of message exposure, seeking and sharing information about e-cigarettes across media platforms. *Tobacco Control.* 2014;23:17-25.

21. Lippert AM. Do Adolescent Smokers Use E-Cigarettes to Help Them Quit? The Sociodemographic Correlates and Cessation Motivations of U.S. Adolescent E-Cigarette Use. *Am J Health Promot.* 2015 Jul-Aug;29(6):374-9.

22. Richardson A, Pearson J, Haijun X, et al. Prevalence, Harm Perceptions, and Reasons for Using Noncombustible Tobacco Products Among Current and Former Smokers. *American Journal of Public Health.* 2014;104(8):1437-44.

23. Camenga DR, Kong G, Cavallo DA, et al. Alternate tobacco product and drug use among adolescents who use electronic cigarettes, cigarettes only, and never smokers. *J Adolesc Health.* 2014;55(4):588-91.

24. Camenga DR, Delmerico J, Kong G, et al. Trends in use of electronic nicotine delivery systems by adolescents. *Addict Behav.* 2014;39(1):338-40.

25. Choi K, Forster J. Characteristics associated with awareness, perceptions, and use of electronic nicotine delivery systems among young US Midwestern adults. *American journal of public health.* 2013;103(3):556-61.

26. Czoli CD, Hammond D, White CM. Electronic cigarettes in Canada: Prevalence of use and perceptions among youth and young adults. *Canadian Journal of Public Health.* 2014;105(2):e97-e102.

27. Grana RA, Popova L, Ling PM. A longitudinal analysis of electronic cigarette use and smoking cessation. *JAMA Intern Med.* 2014;174(5):812-3.

28. Hayes RB, Scheuermann TS, Resnicow K, et al. POS3-160 Smoking and quitting history characteristics among current electronic cigarette users in a national multi-ethnic adult smoker sample. In: SNRT. *Proceedings of the 20th annual meeting of the Society for Nicotine and Tobacco Research; 2014 Feb 5-8; Seattle, USA.*

29. Hyland A, Delmerico J, Cummings M, et al. PA10-4 Use of electronic nicotine delivery systems by teenagers in a longitudinal study In: SNRT. *Proceedings of the 19th annual meeting of the Society for Nicotine and Tobacco Research; 2013 March 13-16; Boston, USA.*

30. Li J, Bullen C, Newcombe R, et al. The use and acceptability of electronic cigarettes among New Zealand smokers. *N Z Med J.* 2013 May 31;126(1375):48-57.

31. Pokhrel P, Little MA, Fagan P, et al. Electronic cigarette use outcome expectancies among college students. *Addict Behav.* 2014;39(6):1062-5.

32. Pokhrel P, Fagan P, Little MA, et al. Smokers who try e-cigarettes to quit smoking: findings from a multiethnic study in Hawaii. *American journal of public health.* 2013;103(9):e57-62.

33. Sherratt FC, Robinson J, Marcus M, et al. E-cigarette usage within a local stop smoking service. Presentation at: *UK National Smoking Cessation Conference*; *2014 June 12-13; London, UK*. www.uknscc.org/uknscc2014_presentation_319.php

34. Sutfin EL, McCoy TP, Morrell HER, et al. Electronic cigarette use by college students. *Drug and Alcohol Dependence.* 2013;131(3):214-21.

35. Vickerman KA, Carpenter KM, Altman T, et al. Use of Electronic Cigarettes Among State Tobacco Cessation Quitline Callers. *Nicotine Tob Res.* 2013;15(10):1787-93.

36. European Commission. *Attitudes of Europeans towards tobacco.* EC, 2012. http://ec.europa.eu/health/tobacco/docs/eurobaro_attitudes_towards_tobacco_2012_en.pdf

37. Welsh Government. *Exposure to secondhand smoke in cars and e-cigarette use among 10-11 year old children in Wales: CHETS Wales 2 key findings report*, Welsh Government Social Research 71/2014, 2014. http://dera.ioe.ac.uk/20494/1/140715-exposure-secondhand-smoke-cars-ecigarette-use-among-10-11-year-olds-chets-2-en.pdf

38. ASH Wales. *Young people and the use of e-cigarettes in Wales.* ASH Wales, 2014. http://ashwales.org.uk/assets/factsheets-leaflets/young_people_and_e-cigarettes_in_wales_final_march_2014.pdf

39. Biener L, McInenrny S. POS4-41 Broad appeal of electronic cigarettes in the smoker population contrasts with relatively low appeal of snus. In: SNRT. *Proceedings of the 19th annual meeting of the Society for Nicotine and Tobacco Research; 2013 March 13-16; Boston, USA.*

40. Brown J, West R, Beard E, et al. Prevalence and characteristics of e-cigarette users in Great Britain: Findings from a general population survey of smokers. *Addictive Behaviors.* 2014;39(6):1120-5.

41. Cho JH, Shin E, Moon S-S. Electronic-Cigarette Smoking Experience Among Adolescents. *Journal of Adolescent Health.* 2011;49(5):542-6.

42. Dautzenberg B, Birkui P, Noël M. E-cigarette: a new tobacco product for schoolchildren in Paris. *Open J Respiratory Diseases*. 2013;3:21–4.

43. Dockrell M, Morrison R, Bauld L, et al. E-cigarettes: prevalence and attitudes in Great Britain. *Nicotine Tob Res.* 2013;15(10):1737-44.

44. Kinnunen JM, Ollila H, El-Amin SE, et al. Awareness and determinants of electronic cigarette use among Finnish adolescents in 2013: a population-based study. *Tob Control.* 2015;24:e264–70.

45. Kralikova E, Novak J, West O, et al. Do e-cigarettes have the potential to compete with conventional cigarettes?: A survey of conventional cigarette smokers' experiences with e-cigarettes. *Chest.* 2013 Nov;144(5):1609-14.

46. Krishnan-Sarin S. E-cigarettes and flavors: Perceptions and use among adolescents and young adults. In: SNRT. *Proceedings of the Society for Nicotine and Tobacco Research Europe Annual Meeting; 2014 Sep 18-20; Barcelona, Spain.*

47. Kvaavik E, Lund M. O-55. Use of e-cigarettes and smokeless tobacco as smoking cessation aids – do they differ by gender? In: SNRT. *Proceedings of the Society for Nicotine and Tobacco Research Europe Annual Meeting; 2014 Sep 18-20; Barcelona, Spain.*

48. Martinez-Sanchez JM, Ballbe M, Fu M, et al. Electronic cigarette use among adult population: a cross-sectional study in Barcelona, Spain (2013-2014). *BMJ Open.* 2014;4(8).

49. Pearson J, Richardson A, Niauras R, et al. POS5-96 E-cigarette awareness, use and risk perceptions among current and former smokers. In: SNRT. *Proceedings of the 17th annual meeting of the Society for Nicotine and Tobacco Research; 2011 Feb 16-19; Toronto, Canada.*

50. Sochor O, Kralikova E, Cifkova R, et al. Tobacco use and some characteristics of tobacco users. Preliminary results of "Kardiovize Brno 2030". *Cor et Vasa.* 2014;56(2).

51. Stillman FA, Soong A, Zheng LY, et al. E-cigarette use in air transit: self-reported data from US flight attendants. *Tob Control* 2014. Published Online First: 20 Jun 2014. doi:10.1136/tobaccocontrol-2013-051514

52. World Health Organisation. *Global adult tobacco survey: Qatar factsheet*. WHO, 2013. http://www.emro.who.int/images/stories/tfi/documents/FACT_SHEETS/FS_GATS_Qatar_2013.pdf?ua=1

53. King BA, Alam S, Promoff G, et al. Awareness and Ever-Use of Electronic Cigarettes Among U.S. Adults, 2010-2011. *Nicotine & Tobacco Research.* 2013;15(9):1623-7.

54. Action on Smoking and Health. Factsheet: Use of e-cigarettes continues to rise among British adult smokers but use among young people is negligible. ASH, 2013. www.ash.org.uk/media-room/press-releases/:use-of-e-cigarettes-continues-to-rise-among-british-adult-smokers-but-use-among-young-people-is-negligible

55. Baeza-Loya S, Viswanath H, Carter A, et al. Perceptions about e-cigarette safety may lead to e-smoking during pregnancy. *Bull Menninger Clin.* 2014;78(3):243-52.

56. Dockrell M. E-cigarettes and shisha: Gateways to regular smoking among British youth. Presentation at: *UK National Smoking Cessation Conference*; *2014 June 27-28; London, UK*. www.uknscc.org/uknscc2013_presentation_261.php

57. Johns M, Shelley D, Farley SM, editors. POS4-18 Predictors of electronic cigarette use among a sample of new york city adolescents. In: SNRT. *Proceedings of the 20th annual meeting of the Society for Nicotine and Tobacco Research; 2014 Feb 5-8; Seattle, USA.*

58. Yong H, Borland R, Balmford J, et al. POS4-46 Changes in e-cigarette awareness, trial, use and relative harm beliefs among current and former smokers in four high-income countries. In: SNRT. *Proceedings of the 20th annual meeting of the Society for Nicotine and Tobacco Research; 2014 Feb 5-8; Seattle, USA.*
